# Electronic Swallowing Intervention Package to Support Swallowing Function in Patients With Head and Neck Cancer: Development and Feasibility Study

**DOI:** 10.2196/formative.9703

**Published:** 2018-08-17

**Authors:** Julie Cowie, Sally Boa, Emma King, Mary Wells, David Cairns

**Affiliations:** ^1^ Nursing, Midwifery and Allied Health Professions Research Unit Glasgow Caledonian University Glasgow United Kingdom; ^2^ Strathcarron Hospice Denny United Kingdom; ^3^ Nursing, Midwifery and Allied Health Professions Research Unit Faculty of Health Sciences and Sport University of Stirling Stirling United Kingdom; ^4^ Nursing Imperial College Healthcare NHS Trust London United Kingdom; ^5^ Department of Computing Science and Maths University of Stirling Stirling United Kingdom

**Keywords:** head and neck cancer, eHealth, self-management, mHealth, chemoradiotherapy, mobile phones

## Abstract

**Background:**

Many patients undergoing treatment for head and neck cancer (HNC) experience significant swallowing difficulties, and there is some evidence that swallowing exercises may improve outcomes, including quality of life. This feasibility study developed an evidence-based, practical Swallowing Intervention Package (SiP) for patients undergoing chemoradiotherapy (CRT) for HNC. As part of the study, an electronic version of SiP (e-SiP) was concurrently developed to support patients to self-manage during treatment. This paper reports on the e-SiP component of this work.

**Objective:**

The objective of our study was to develop and conduct a preliminary evaluation of an electronic support system (e-SiP) for patients undergoing CRT for HNC.

**Methods:**

The study was conducted using a recognized mHealth development and evaluation framework and involved health professionals and patients who were undergoing CRT for HNC. The scoping stage of e-SiP development investigated the potential usefulness of the app, exploring how e-SiP would look and feel and what content would be appropriate to provide. Patient and carer focus groups and a health professionals’ consensus day were used as means of data gathering around potential e-SiP content. A repeat focus group looked at an outline version of e-SiP and informed the next stage of its development with regard to refining the requirements for the tool. This was followed by further development and a testing stage of e-SiP that involved the coding of a prototype, which was then evaluated using a series of steering group meetings, semistructured interviews with both patients and health care professionals, and analysis of e-SiP log data.

**Results:**

Feedback from focus groups and health professional interviews was very positive, and it was felt e-SiP use would support and encourage patients in conducting their swallowing exercises. However, of the 10 patients who were offered e-SiP, only 2 opted to use it. For these patients, the aspects of the e-SiP app were considered useful, in particular, the ease of keeping a diary of exercises performed. Interviews with users and nonusers suggested significant barriers to its use. Most significantly, the lack of flexibility of the platform on which e-SiP could be accessed appeared a dominant factor in deterring e-SiP use.

**Conclusions:**

The results suggest that further research needs to be conducted around the implementation of e-SiP. This involves evaluating how e-SiP can be better integrated into usual care and through patient training and staff engagement, can be perceived as a beneficial tool to help support patients in conducting swallowing exercises.

## Introduction

There are an estimated 400,000-600,000 new cases of head and neck cancer (HNC) globally each year [1]. In the United Kingdom (UK), approximately 11,000 people are diagnosed with HNC annually, making it the eighth most common cancer. In Scotland, the rates of HNC are almost 40% higher than those in England [2]. There is a link between HNC and the presence of the human papillomavirus (HPV), and HPV-positive cases now account for around 30%-65% of HNCs [3]. The demographics of HNC are therefore changing because patients who are positive for HPV tend to be younger at diagnosis generally have a higher socioeconomic status and better education and a better prognosis (despite often presenting at a more advanced stage of cancer) than patients with HPV-negative HNCs [3-5]. Younger age at diagnosis and improved treatment effectiveness mean that more people are now living with the consequences of HNC and its treatment.

Treatment for HNC can include a combination of surgery, radiotherapy, and chemotherapy [6], leading to both acute and chronic adverse effects. The site of the tumor and the side effects of treatment can impact eating and drinking, physical appearance, and communication [7,8]. Improved treatments have lowered mortality rates but at the expense of greater morbidity with many patients experiencing long-term or permanent swallowing problems (dysphagia) [9] and younger survivors reporting the most severe problems [10]. Preparing patients for potential swallowing problems is clinically advised, but it is unclear when and how this should be done [11].

There is emerging evidence that giving patients prophylactic swallowing exercises may improve long-term swallowing outcomes for HNC patients [12]. These exercises target the swallowing muscles to strengthen and maintain the normal range and speed of swallowing movements and increase blood flow to muscles, which may reduce or prevent fibrosis [13,14]. Trial results are mixed [15], however, and questions remain about the most effective type of exercises, the dose, the most optimal time of introduction, and how best to support patients in adhering to the exercises [14]. Only 13%-14% of participants practice swallowing exercises as recommended [16,17], although how to effectively measure adherence to swallowing exercises is unclear, especially because the optimal dose of exercise is often unknown [18]. There is evidence that those able to maintain their exercise schedule achieve improved swallowing outcomes [19] and are less likely to need a feeding tube [20].

A number of commercial mobile apps have now been developed to support people with dysphagia, and there is anecdotal evidence that these are used and valued by speech and language therapists (SLTs) in clinical practice. It has been suggested that mHealth technology should be developed in partnership with stakeholders and tailored to the unique needs and experiences of the specific population it seeks to support [21,22]. Many of the apps currently in use are generic, rather than being developed for use with a specific population. To date, little empirical research has been carried out to evaluate the effectiveness or the extent of their use with the HNC population. Starmer et al [23] conducted a feasibility study to explore the use of an app for patients undergoing radiation-based treatment for HNC. Our research complements and extends this work by involving patients, carers, and clinicians in the design and development of a suitable tool to support swallowing function.

This paper reports on research that was undertaken to develop and evaluate an electronic Swallowing Intervention Package (e-SiP) for patients undergoing chemoradiotherapy (CRT) for HNC. The tool aims to support patients to conduct swallowing exercises to improve long-term swallowing function and quality of life. The work was performed as part of a larger feasibility study to develop and test a paper-based SiP for the above patient group [24]. In the remainder of this paper, we describe the work undertaken in the development and testing of e-SiP, which involved an initial scoping exercise, development of the tool, and a preliminary evaluation. Findings are presented followed by reflections and concluding comments on how this research might be used to inform future studies.

## Methods

### Overview

**Figure 1:**
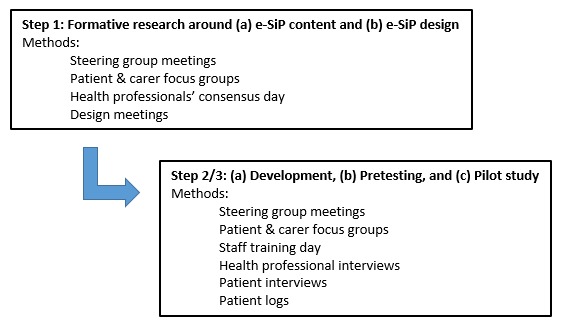
Electronic Swallowing Intervention Package (e-SiP) development and evaluation process.

The development and feasibility testing of e-SiP were conducted following the development and evaluation framework proposed by Whittaker et al [25]. This framework highlights the importance of a staged approach to mHealth apps to ensure that the development and evaluation process is rigorously conducted. Our paper focuses on the early steps of the process: the conceptualization of e-SiP, conducting formative research using a number of group meetings with relevant stakeholders, and pretesting and piloting the prototype system. Figure 1 depicts the data collection methods used at each stage.

Recognizing the importance of user-centered design in the development of any mHealth app, our work has also drawn on social cognitive theory [26] to explore how individuals acquire information and use this to influence behavior. In addition, the work of Cooper et al [27] around patients’ beliefs and its impact on adherence has underpinned discussions about the design of the e-SiP app.

### Step 1: Electronic Swallowing Intervention Package Formative Research

Questions about the key components of a feasible and evidence-based e-SiP for HNC patients undergoing CRT were incorporated into a series of events and meetings held to gain views from clinicians, patients, and carers. This scoping exercise comprised patient and carer focus groups, a health professionals’ consensus day, and steering group committee meetings. The purpose of these meetings was to elicit appropriate content to include in e-SiP and how the tool should look and feel. Feedback from these discussions were used in a series of design meetings held with software architects.

### Steps 2 and 3: Electronic Swallowing Intervention Package Development, Pretesting, and Pilot Study

An initial version of e-SiP was developed, and this prototype was demonstrated and discussed at a further series of events (clinical staff training day, steering group, and a patient focus group) to clarify that the content and design was acceptable and usable for clinicians and patients. This participatory approach ensured that the views of a range of stakeholders were incorporated into the software design prior to the final version of e-SiP being trialed. In addition, an e-SiP user guide was developed and presented to staff along with the training to further support them in using the system and teaching patients wanting to use e-SiP. This iterative process of codesign was crucial in ensuring that the tool met user needs.

e-SiP was offered to study participants, and its use was evaluated through electronic logs and semistructured interviews with patients (both e-SiP and paper-based SiP users) and health professionals. Eligible patients were approached by an SLT or clinical nurse specialist (CNS) in their local health board, and the SiP study was explained to them (Table 1). If a patient indicated that they might be interested, they were provided with information about the study and gave verbal consent for the research nurse team to contact them in a few days’ time. If they opted to take part, consent and baseline data collection were undertaken by the local research nurse team. Participants in the health board that trialed e-SiP had the additional option of using an iPad loaned by the study to access e-SiP. Digital literacy was not examined, and all patients were offered the use of an iPad regardless of their previous experience. All patients were given the option of consenting to be contacted regarding a later qualitative interview and were given information sheets to pass to their carers, should they also wish to be interviewed.

#### Outcome Measures

Patient- and clinician-reported questionnaires were completed at baseline (before the intervention started), at the end of CRT, and 3 and 6 months after the end of CRT. These included the water swallow test (not performed at the end of CRT) and MD Anderson Dysphagia Inventory measures of swallowing, performance status scale for HNC patients, and the Functional Oral Intake Scale. Measures of quality of life were the EuroQol EQ-5D-3L, the EORTC Quality of Life Questionnaire-C30 with the additional Head and Neck Cancer Module HN37, and the Brief Illness Perception Questionnaire.

The questionnaires were completed during appointments with a research nurse or via post if the patient was too ill to attend. Both the patients using e-SiP and the paper-based SiP completed the questionnaires on paper.

**Table 1:** Main components of Swallowing Intervention Package.

Time of intervention	Type of support provided	Details of support provided
Pretreatment intervention: One-on-one consultation with SLT^a^ (with carer present as desired and appropriate).	Discuss Swallowing Intervention Package folder (or electronic Swallowing Intervention Package if selected); demonstrate swallowing exercises.^b^	Instruct patient about the importance of practicing swallowing daily. Instruct the patient about how to do swallowing exercises including a demonstration. Increase patient motivation to complete swallowing exercises. Help patient plan swallowing exercises and overcome barriers. Help patient to set long-term goals.
Weekly review during radiotherapy: Consultation with SLT or clinical nurse specialist as part of usual care.	Reinforce intervention; complete weekly assessment sheet: Monitor symptoms and pain management, and record behavior change techniques used.	Check homework and review goals. Motivate and encourage patient to complete swallowing exercises and diary record sheets. Address any issues which have arisen. Provide additional demonstration of exercises if requested.

^a^SLT: speech and language therapist.

^b^Effortful swallow, Masako maneuver, Mendelsohn maneuver, Shaker head-lifting maneuver, and jaw exercises.

#### Analysis

Log data detailing the patients’ use of e-SiP were analyzed using SPSS (IBM Corp, Armonk, New York, USA). Transcripts of the interviews and focus groups conducted were analyzed using a thematic framework approach [28] and coded with the support of NVivo 10 software (QSR International, Melbourne, Victoria, Australia). The initial framework was devised both inductively and deductively. Field notes were used to capture discussions on the consensus day, training day, and steering group meetings. These were analyzed, and the data were structured around emerging themes.

## Results

### Step 1: Electronic Swallowing Intervention Package Formative Research

#### Step 1a: Electronic Swallowing Intervention Package Content

The content of e-SiP was developed through an analysis of the literature and discussions with patients and health professionals, as outlined below. The aim was to make it a tool that was regarded as patient-focused, practical, and evidence-based.

##### Patient and Carer Focus Groups

Four initial focus groups were carried out across two health boards (one of which was the health board in which the SiP was to be trialed). In total, 23 people participated (16 patients, 7 carers). The experiences discussed by focus group participants helped us to identify the most important information to include in the e-SiP package. Using the thematic framework approach, 3 researchers were involved in coding, cross coding, and sense checking of the data collected. The emerging themes from the data are discussed below.

###### Effects of Treatment

Participants spoke of the pain associated with the side effects of treatment, the long recovery time (for which many felt unprepared), and the effort and time required for eating and drinking as their swallowing deteriorated. Difficulties around eating included discomfort, dry mouth, changes in taste, the requirement for a softer diet, and fatigue, which made eating a chore. Many of the patients had used a feeding tube during their treatment. Even months after the end of treatment, many patients still experienced swallowing difficulties with 2 patients being unable to swallow anything at all.

I was dehydrated because I had stopped eating, I’d stopped drinking and it was physically impossible for me to swallow, the pain was just unbearable.

Patient

...well, basically to grind...I can't eat meat. Oh, to have a steak, would be brilliant. To have chips would be brilliant. Although, it has helped my weight. But no, I can't chew, so it's pasta, fish. I'm adapting, you know, to chewing.

Patient

###### Need for Clearer Explanation of Possible Swallowing Difficulties

Participants reported that although clinical staff had discussed potential swallowing problems with them, they had found this difficult to grasp without any previous experience of what swallowing problems could be like. Participants stated that it would be useful to hear about experiences from other patients who had been through treatment because for many patients, this was the first time that they had met others who had undergone HNC treatment. Firsthand experiences and accounts from previous patients were, therefore, included in the e-SiP resource. Focus group participants also felt that information on preparing food and understanding more about how the swallowing muscles worked would be useful to include in the package.

###### Need for Clearer Description of Recommended Exercises

Some focus group patients had been given a sheet of exercises during their treatment but with little explanation, and others had not been given any exercises. The participants suggested that for e-SiP, access to both written instructions and videos of the exercises would be useful. They also liked the idea of having access to information and support to motivate them and manage the psychological challenges of going through treatment for HNC.

Yes, I must admit I didn'tknow if I was doing them [exercises] right and how long to have them for and things like that so [videos of exercises] would be useful.

Patient

You could do them without thinking. Then you just have a wee bit, oh they're doing that and then you try it for a wee while and again, right oh they're doing that one, they're doing this one, and you're still watching the telly. I used to do mine watching the football and sit there and the wife’s going, what are you doing? I've been doing my exercises. But, whether I was doing them right is another thing, or long enough is another one.

Patient

###### Need for a Diary to Support Tracking of Progress Performing the Exercises

It was also decided that e-SiP should feature an electronic diary in which patients could mark down how many exercises they had managed to achieve in a day or even just record their eating habits over a period of time. Patients felt that this would help them track progress, keep motivated to continue conducting the exercises, and give back some control over their illness.

I think breaking it down is very, very, important and when you're probably at your bleakest and at your tiredest, you wouldn't want to fill things in or write things down. But,...[filling in a diary] it's giving you control again to your illness, it's giving you ownership of your condition.

Patient

##### Health Professional Consensus Day

Nineteen clinical and academic staff attended a consensus day where the content of the paper-based package and e-SiP was agreed. All of the swallowing exercises proposed for inclusion in e-SiP are already in clinical use, but the delivery and instructions given vary between different health boards. The standard package of exercises for e-SiP was therefore decided upon using a consensus exercise with SLTs. Exercise instructions were gathered from health boards involved in the e-SiP study (or in which the advisors worked) and rewritten or redrawn to make a consistent package that could be used for e-SiP. Attendees also provided input into additional health information e-SiP would feature, such as keeping the mouth clean, managing mood and anxiety, and suggestions for eating a modified diet. A link to the Macmillan website discussing nutrition in cancer was also suggested.

There is a significant body of research on health communication and the importance of tailoring information to an individual’s needs [29,30]. In many apps, tailored health information has been seen to increase the effectiveness of the message [31,32]. As such, discussion at the consensus day also focused on how e-SiP could be personalized to individual patients. It was decided that the videos of individuals performing the exercises would be a useful inclusion. Rather than using generic videos already available online, to personalize and customize the information to the individuals who would use e-SiP, new recordings of the videos by UK-based SLTs were produced. A further request regarding the swallowing exercises was that only exercises relevant to a particular patient be included for that particular patient. Again, this ensured that e-SiP was highly tailored to individuals in an attempt to encourage its relevance and subsequent use by participants.

##### Steering Group

Our steering group comprised 13 clinicians, 3 academics, and 3 patient advisors, who gave us invaluable information about their experiences of going through treatment for HNC. The patient advisors read through all the proposed e-SiP information to make sure that it was suitable and accessible to the patients in our study. Additional features were also proposed, including an assessment of barriers to performing the exercises (symptoms and time), an email facility, and the ability to record a video diary.

##### Final Electronic Swallowing Intervention Package Content

The content of the e-SiP can be categorized as follows: study information; exercise videos; exercise information; HNC and treatment information; audio and written information on managing anxiety and low mood; diary, calendar, and email facility; video diary; and patient stories. Many of these features overlapped with those provided with the paper-based SiP. However, there were perceived to be certain advantages over the paper copy: (1) e-SiP would include direct links to external sites with information about HNC, whereas we could only provide the website address for the paper-based SiP; (2) for the exercise diaries, e-SiP would include an automatic recording of when the diary was filled in and would also prevent patients filling in diaries for the previous days, an aspect that could not be fulfilled by the paper-based version. We hoped that this would give us a good comparison to assess if hoarding occurred with patients using the paper diaries, as has been reported elsewhere [33-35]; and (3) e-SiP would contain videos of exercises, recordings of coaching exercises for managing anxiety and low mood, and clips of former patients discussing eating and drinking. The provision of this information in the paper-based version of SiP was via a digital video disc.

#### Step 1b: Electronic Swallowing Intervention Package Look and Feel

A series of 6 design meetings were held with key researchers on the project and the e-SiP app design team. These meetings were held over a 6-month period and involved an iterative process of design, reflection, and development. Initial ideas for e-SiP were developed using a storyboard approach, mapping out how users might progress through the app. The storyboard constructed to represent interaction with e-SiP is shown in Multimedia Appendix 1. Having finalized the storyboard, work began to translate this into a usable prototype app.

### Steps 2 and 3: Electronic Swallowing Intervention Package Development, Pretesting and Pilot Study

#### Steps 2 and 3a: Electronic Swallowing Intervention Package Development

e-SiP was developed using Apple’s XCode Integrated Development Environment and is written in Objective-C. It makes use of locally stored media, including web content and video, and enables users to save their video recordings and access remote content relevant to the SiP project (eg, the Macmillan Cancer Support website). The current version of e-SiP is compatible with Apple iPad 2s, and it is via this medium that the app was made available for user testing. Currently, the app is not available for any other operating system, and it is not available on Apple’s App store for download onto an individual’s device.

In line with the storyboard developed as part of the Phase I exploration, e-SiP provides users with an easy-to-use, simple interface through which the variety of services it offers can be accessed. Multimedia Appendix 1 depicts e-SiP’s main screen through which all the functionality available can be accessed. The interface is very much in keeping with the current style of interface offered by Apple and is generally regarded as intuitive to use. However, testing of the prototype’s usability, as well as the suitability of content and ease of navigation, was conducted at the pretesting phase of the project.

The main screen is divided into four main sections: study information, swallowing exercise videos, exercise instructions, and patient stories. These sections allow the patient to access information about each area described. These four information sections are accompanied by a menu bar at the bottom of the screen. This menu is designed such that the user can keep track of the exercises performed as well as record information about their symptoms, keep a diary relating to how they felt on a particular day and their experiences of the exercises, and use the calendar function to track hospital appointments and important dates. The settings component of the menu bar allows e-SiP to be tailored to an individual patient; for example, when giving e-SiP to a patient for the first time, SLT can specify what exercises should be provided for the specific patient, ensuring only exercises appropriate to the patient’s needs are shown on e-SiP’s main screen.

The recording of calendar entries whereby patients can detail exercises completed each day along with information relating to their experience of conducting these exercises is depicted in Multimedia Appendix 1. In the example given, the patient has created 6 entries on the September 17 and 2 entries on the September 18.

Along the top of the calendar screen is a further option bar allowing users to “Add Event,” “Record Exercise Reps,” “Exercise Feedback,” and “Send Email.” The “Record Exercise Reps” option allows the user to record details of exercises completed. The “Exercise Repetitions” screen is shown in Multimedia Appendix 1.

As explained in the instructions at the top of the screen, the user should select which session they are completing, after which they should use the slide bar to select how many of each exercise they completed. If the user was unable to complete any exercises in a given session, they could move the slide button to the right next to the option “I was not able to complete any of the exercises” to indicate that none were completed.

The “Exercise Feedback” option allows the user to record whether anything interfered with them in conducting their exercises. This should be completed daily. For each option listed, the user should indicate how much (from “not at all” to “very much”) each option made it more difficult for them to complete their exercises. In the example shown in Multimedia Appendix 1, this user had no pain or discomfort, was moderately tired, was feeling very low, and felt they didn’t have much time.

#### Steps 2 and 3b: Electronic Swallowing Intervention Package Pretesting

Prior to evaluating e-SiP with patients, the tool was demonstrated in a number of settings to receive and act on feedback regarding its usability and potential for incorporation into care provision. e-SiP was trialed and evaluated by participants in a repeat focus group (2 patients, 1 carer), at a clinicians’ training day (n=19), and steering group meetings (n=18). Feedback from these meetings was extremely positive and indicated that e-SiP had the potential to support patients in conducting swallowing exercises and monitoring their progress. Some of the key themes arising from the meeting were that e-SiP was felt to be something patients would benefit from (patient focus group) and that the exercise videos were an extremely useful component (patient focus group, staff consensus day).

See, when I tried the Mendelsohn maneuver, and actually, you can do it wrong as well as right. I mean, if you just lift...you can lift without swallowing. But if you don't swallow, there's no point doing it. And it's very easy done. And it was described to me, and it was described to me in about three and a half seconds, and that was it. And I was given a piece of paper, but the piece of paper, it's not bad. But a video, that's the answer.

Patient, focus group

Furthermore, patients would engage with the tool as it felt “local” and relevant to those receiving treatment as part of the National Health Service (NHS) system (steering group, SLT). Lastly, patient experience stories are a useful inclusion because it was felt that patients are more likely to listen to other former patients than a clinician (steering group, patient advisor).

#### Steps 2 and 3c: Trialing Electronic Swallowing Intervention Package With Patients

Although the wider SiP study was conducted in 5 NHS boards across the UK, e-SiP was only trialed in 1 health board owing to complexities around gaining information technology (IT) governance within the timescales of the study. e-SiP was offered to 10 participants with 2 choosing to use it. As reported in more detail in the qualitative interviews below, most of those who declined e-SiP were simply happy with the paper copy. The very small numbers preclude any analysis of demographic or clinical factors that may have influenced the uptake of e-SiP. Both participants who opted to use e-SiP were men aged 54 and 62 reflecting the demographics of the SiP cohort as a whole. Both lived in postcode areas coded as level 4 on the Scottish Index of Multiple Deprivation (SIMD) [36], which was slightly higher (more affluent) than the median SIMD. The log of e-SiP use by the 2 users was analyzed (see below). Both patients using e-SiP took part in qualitative interviews along with 15 other study participants purposively sampled to represent people of different ages, diseases, and treatment characteristics. They were also asked their views about the potential use of e-SiP. Additional feedback from health professionals was gained through qualitative interviews.

Patients using both e-SiP and the paper-based version of SiP were asked to start exercises and diary record cards on their first day of radiotherapy and continue logging their exercise achievements daily throughout their course of radiotherapy (approximately 6 weeks). Posttreatment, patients were advised to stop the exercises when SLT agreed that their swallowing levels had returned to an acceptable level. Patients on the paper version were provided with diary cards for around 1 month posttreatment. However, most had stopped logging their exercises by 2 weeks posttreatment. All questionnaires (eg, quality of life questions) carried out as part of the trial were given on paper, regardless of whether the patient was an e-SiP or a paper-based SiP user.

##### Summary Electronic Swallowing Intervention Package Log Data

From the log data recorded from patients’ use of e-SiP, we were able to see what aspects of the system were used by patients and how often. Both patients used the diary facility each day. In addition, they recorded daily feedback relating to how they were feeling, how easy (or not) the exercises were to complete, and which exercises proved to be more challenging than others. One patient used e-SiP to record his symptoms each day. Both patients viewed the videos showing how to conduct the various recommended exercises at the beginning of their treatment. They also viewed the film clips of people talking about their experiences of eating and drinking after the HNC treatment, and they looked at the “Managing Worries” and “Useful Links” sections. All of this activity took place at the beginning of treatment. Once they had started carrying out the swallowing exercises regularly, they no longer looked at the other materials. Neither participant used the video diary facility.

Given that only 2 patients opted to use e-SiP, it is not possible to draw any conclusions from the data about which components were most useful, although the use did appear to mirror how people used the paper version (mostly for recording exercises and how they were feeling).

##### Feedback From Patient Interviews

The 2 participants who used e-SiP were interviewed and had the opportunity to talk about their experiences of using it. Ten participants (of the 15 interviewed) who had used paper-based SiP also talked about the potential use of e-SiP. The following themes arose from the interviews.

###### Ease of Use

Participants who used it found e-SiP easy to use and had no problems navigating the system:

Aye, great, aye, no problem at all, very self-explanatory, basically.

T004, e-SiP user

However, both participants found it frustrating that they had to record which exercises they had done on the specific day rather than completing it retrospectively:

I remember, in actual fact, it was literally on, I think, I'm pretty sure it was on the stroke of midnight. Because, you know, if we were late or something, and then, I'd go at quarter past midnight, or something, and you would go, oh no, it wouldn't let you do it. It was just so blooming frustrating.

T010, e-SiP user

Both participants completed the diary entries once a day rather than as they did the exercises.

###### Use of Own Technology

Participants who used e-SiP would have preferred to have been able to use it as an app on their own phone or tablet rather than having to use an extra piece of technology:

Some people would find that easier to carry, you've always got your phone on you. So, it would probably be something that you could take into the hospital, and you could sit while you were getting your chemo, and your different thing, rather than carrying a big iPad about with you. Yeah, that would probably be quite a good idea.

T004, e-SiP user

But one less bit of technology to have about, probably, would have been helpful. Or even if the app could have been downloaded onto my iPad, do you know what I mean?

T010, e-SiP user

###### Video Diaries

Neither participant who used e-SiP used the video diary function and did not appear to be very interested in this as a feature:

I'm not very good with that stuff, to be honest.

T004, e-SiP user

###### Video Material

One participant found the film clips of people demonstrating the swallowing exercise helpful:

Well that's probably, I found that [watching videos] more helpful than reading the literature...because you actually, they were showing you exactly what to do, and you were getting into the way of it better.

T004, e-SiP user

The same participant engaged in the Web-based materials and found them useful, particularly being able to hear about other people’s experiences:

But again, if you read that, and then you have a look at the videos and you can see, okay, this is what I have, and that one is a lot bigger than mine, you know, the growth, and some people could hardly even swallow. Mine was quite small, it was like a little bit of popcorn, you know, that's what it looked like, or a nugget, kind of thing, one of these nugget things.

T004, e-SiP user

A small number of patients who had not used e-SiP were ambivalent about the videos, 2 specifically saying that they thought that the written information was sufficient. However, others wanted to be able to see (through diagrams and video) whether they were doing the exercises correctly.

###### Reasons for Opting for Electronic Swallowing Intervention Package

One participant chose to use the iPad because he was accustomed to using it on a daily basis and felt that it helped him to remember to use it:

I suppose, I have an iPad, so I'm used to using the iPad. And it's more likely, if I'm using the iPad every day, that I'll, at some point during the day, I'll be reminded that I need to update the information on that iPad.

T010, e-SiP user

Two participants who had not used the e-SiP felt that they would have found it useful because one was accustomed to using their iPad regularly. Another felt that it would have reduced the amount of paper around the house. Most of the others interviewed appeared to be more comfortable with the paper-based SiP, saying things such as:

I’ve got a computer, I could’ve used it but I prefer this...I like being able to flip backwards and forwards.

L012

Overall, participants were ambivalent about having a choice between the electronic and paper copy of SiP with some preferring the paper version and others saying that they may have opted for e-SiP if they had been able to download it onto their own device.

##### Feedback From Health Professional Interviews

Overall, 15 health professionals involved in the delivery of the SiP intervention were interviewed about their experiences with some questions asked about the e-SiP app. Key findings from these interviews are summarized below.

###### Why Participants Opted for Swallowing Intervention Package (Not Electronic Swallowing Intervention Package)

Staff were surprised that only 2 participants used e-SiP and discussed the possible reasons for this:

No, and in terms of the iPads I’m not really sure why people have been reluctant because...Okay, we missed the first couple because we didn’t have them but they’ve been offered to the majority of people.

SLT1

I cannot believe that nobody, well, not nobody, but hardly anybody took that up, it’s just...and maybe we didn’t sell it enough at this end, I don’t know or maybe it’s the population, maybe if we were...I know [place] is a city, but not many of the patients were actually from [place], quite rural communities that they came from, so whether that makes a difference, I was really surprised.

SLT2

Some professionals felt that participants would have preferred to have used e-SiP on their own phones or iPads:

And I can see that if I was doing it and I had to have a separate iPad rather than it being on my IPad, not that I have an IPad, it gets taken over by everybody else, family IPad, so then maybe you have more ownership of it if it’s on yours as an app, I don’t know if that’s impossible to do with the small scale that we were looking at, I don’t know, I was surprised that nobody wanted it.

SLT2

Others felt that people just preferred a paper-based system:

I did too but actually I think sometimes when people write things down on a bit, I think they feel that actually that’s then...they like the folder because they all came with their folder and all the local cards in their folder, so everything was there for them without having to scroll through an iPad. I think they liked that.

CNS3

###### Logistics

Professionals felt that more participants may have engaged with the technology but because there was a delay in having the iPads ready, not everyone had the option to use e-SiP right from the beginning:

Yeah, because I’m sure [Patient T001] would have used an iPad, but it wasn’t available when she went through…. And she’s very computer literate.

SLT2

###### Knowledge of Technology

Professionals felt that participants’ knowledge of technology varied, and this may have affected their desire to use e-SiP:

I suppose it depends on how computer literate you are. But I did think that would have maybe been easy because you’re not actually having to jot anything down, you’re just hitting buttons if you like.

CNS1 and SLT4, joint interview

I think it would very much depend on the patient and how, kind of, text-savvy they already are.

SLT5

###### Own Technology

Most professionals agreed that more people would have opted for e-SiP if it could have been downloaded onto participants’ own smartphones or tablets:

I think as they’re moving up, I think the population is changing, I think they will all be onto smartphones. I mean, the gentleman that I would never have thought, but that’s a smartphone that he’s just shown me – how good he is at using it I’m not sure, but certainly it was a smartphone he had, and I think over the coming years anyway, the next couple of years, it’s just going to be second nature.

CNS1

And I definitely think our patients are such a diverse group that yeah, some will have them, some won’t.

SLT4

###### Monitoring and Review

In practice, professionals were unsure how to review diary entries on the iPads, and this was problematic for them both in terms of reviewing and monitoring:

No, because you could read in the comments, in the diary comments you could actually read the comments that patients have written so that if somebody for instance had said it was very painful or a struggle, I can go back and say right last week you said this and this, is it better this week? Whereas with the iPad I didn't have access to that.

CNS3

Compliance exactly, compliance as well because somebody can just fill in an iPad to say yes I’ve done it but actually have you really done it? Whereas the diary cards and because they needed to write comments I think for me I think were better.

CNS3

Overall, professionals were surprised by the low uptake and use of e-SiP but concluded that a variety of factors contributed to this, including that they may not have promoted it sufficiently. One clinician, when interviewed, admitted that she had forgotten about e-SiP and, therefore, did not discuss it with any of her patients. This reflects the complexity of the study as a whole because clinicians had many aspects to remember with e-SiP being only one small part of the overall study.

##### Quality of Life Measures

EQ-5D-3L questionnaires asked patients about issues such as mobility, pain and discomfort, and anxiety and depression. Owing to the low numbers of patients using e-SiP and in the control population of the study, it was not possible to draw any definite conclusions about the quality of life changes of patients. However, it appeared that there were potential improvements in self-care and anxiety and depression in the SiP patients.

## Discussion

### Principle Findings

This paper describes the development and preliminary evaluation of an e-SiP designed to support patients with HNC to maintain swallowing function during treatment. Our findings illustrate the potential for involving different stakeholders in the development of a tailored electronic intervention and show that e-SiP offers a practical alternative to a paper-based diary and support system.

However, our experience shows that patients with HNC tend to prefer the paper-based system rather than an electronic app. Patient interviews suggest that having to access the app on a bespoke iPad rather than on a patient’s own phone or tablet was largely responsible for the low uptake of e-SiP. Patient logs illustrate that although the diary feature and videos were well used, other aspects of e-SiP received limited attention. Videos tended to be used during the first few days of a patient’s treatment and less so as the weeks went on (quite possibly reflecting their growing confidence in conducting the exercises correctly).

Staff interviews suggest that the complexity of the study overall [24] meant that clinicians already had a range of responsibilities for data collection and intervention delivery and that they paid relatively little attention to encouraging e-SiP use. Additionally, IT governance systems varied across different NHS sites and created barriers to offering e-SiP to all patients involved in the larger study. This may have also led to e-SiP being presented as an addition to the paper-based version rather than a straight choice between one or the other, which may have driven patients to stick with the status quo of the paper version.

The study highlights a number of issues that would need to be addressed in the future development and adoption of e-SiP. Firstly, it is clear that electronic apps are more likely to be attractive to patients if they can be used on patients’ own devices and are offered on a variety of platforms. However, providing an iPad free of charge to participants in the SiP study eliminated a potential problem of reduced uptake by participants from lower socioeconomic areas who might not otherwise be able to afford the technology [37]. Secondly, mechanisms for sharing e-SiP data between patients and clinicians must be developed so that information and progress can be discussed during consultations. Despite the email facility offered on e-SiP, health care practitioners did not show this feature to users. In addition, one user did make use of the facility, but their corresponding SLT did not check the emails received. Furthermore, if electronic apps are to be integrated into routine practice, IT governance systems need to be more flexible and encouraging of their use. Finally, patients and health care professionals need support and training to use all the features of e-SiP even when they are familiar with the technology. Despite the clinical training and user guide provided, it became apparent from staff interviews that some of the functionality of the app was not used to its full advantage by staff; for example, none of the staff talked about the use of the “back area” of the app to allow them to change goals or add and remove certain exercises for participants. The patients who used the app found it easy to navigate. However, they both were already familiar with the use of technology and iPads, and this is known to make the use of such apps more likely [37,38].

### Study Limitations

This study was conducted to investigate the feasibility of an electronic tool to support swallowing exercises for those living with HNC. The study provided a means of exploring how, through coproduction with patients, carers, and health care professionals, a tailored health tool could be developed. A small pilot of the resulting tool has provided interesting results for the refinement and larger scale testing of such a tool in the future.

It is recognized that given the low numbers of patients that opted to use e-SiP, more extensive testing of the prototype needs to be conducted to draw conclusions about its potential usefulness in promoting swallowing exercises. Our intention is to build on this work and conduct a larger trial of e-SiP considering findings from this study. This work will incorporate more extensive analysis of system log data and recruitment of a larger population of e-SiP users such that further understanding of its potential usefulness can be gained. The study will also make e-SiP more widely available and enable users to freely download it onto their own mobile device.

### Contribution to the Field

This paper provides valuable insight into the potential use of e-SiP technology to support patients to undertake swallowing exercises and manage their swallowing difficulties while they are receiving treatment for HNC. It also provides important lessons for the wider application of technology to support individuals to self-manage and take ownership of their care. In particular, the study highlights the importance of stakeholder involvement in the development of any intervention and the need to address issues of implementation and their potential to impact on the success or failure of an intervention. Initial findings suggest that further evaluation is needed to look specifically at acceptability and usability using “psychometrically robust measures,” as recommended by Darlow and Wen [22].

## Appendix

Multimedia Appendix 1 Electronic Swallowing Intervention Package (e-SiP) storyboard and selected screenshots.

## Abbreviations

CNS: clinical nurse specialist
CRT: chemoradiotherapy
e-SiP: electronic Swallowing Intervention Package
HNC: head and neck cancer
HPV: human papillomavirus
IT: information technology
NHS: National Health Service
SIMD: Scottish Index of Multiple Deprivation
SiP: Swallowing Intervention Package
SLT: speech and language therapist
UK: United Kingdom
