# Supplementary material for: Electronic Swallowing Intervention Package to Support Swallowing Function in Patients With Head and Neck Cancer: Development and Feasibility Study
Source: JMIR Form Res. 2018 Aug 17;2(2):e15. doi: 10.2196/formative.9703 (PMC6181202; doi:10.2196/formative.9703)
Supplement: Multimedia Appendix 1 [file formative_v2i2e15_app1.pdf]

Appendix 1

Figure A1. Storyboard representation of e-SiP

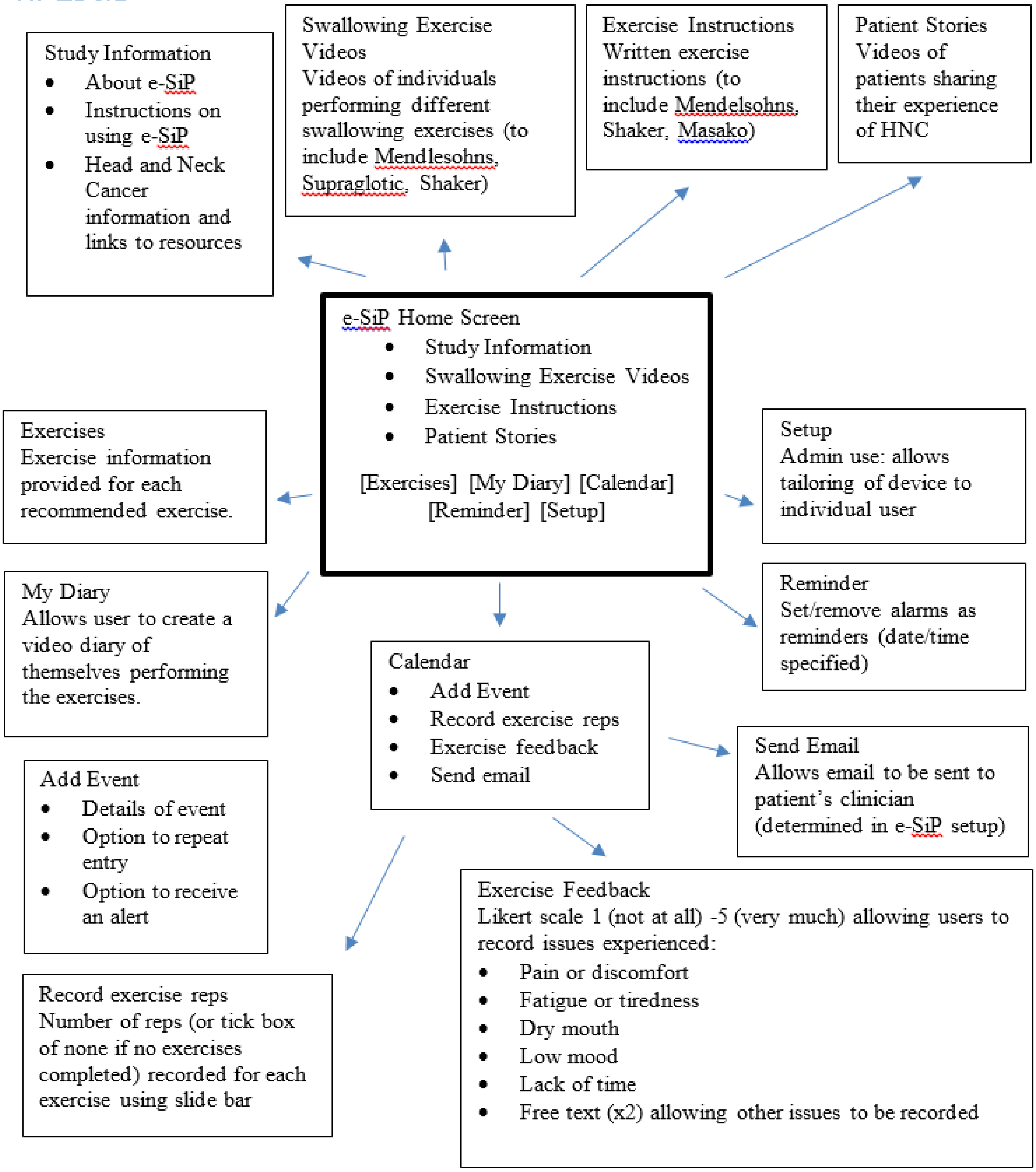

Figure A2. e-SiP main screen

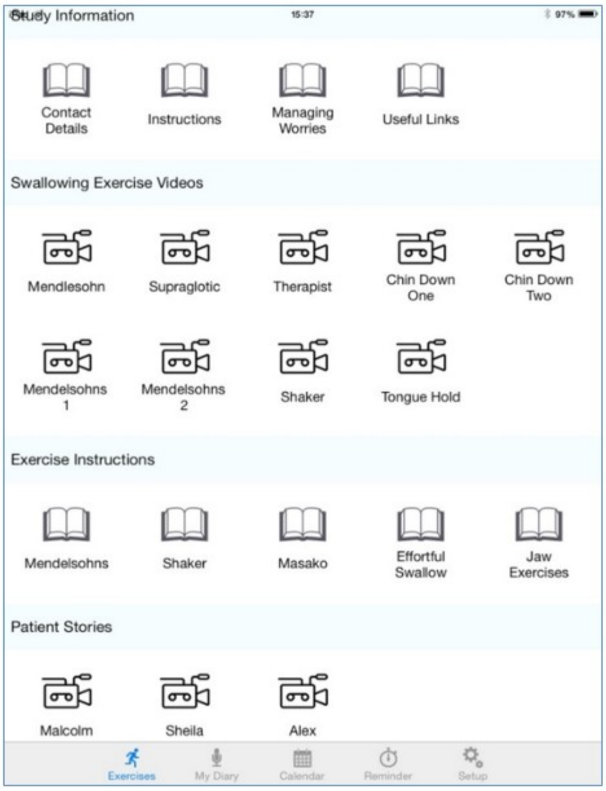

Figure A3. e-SiP calendar

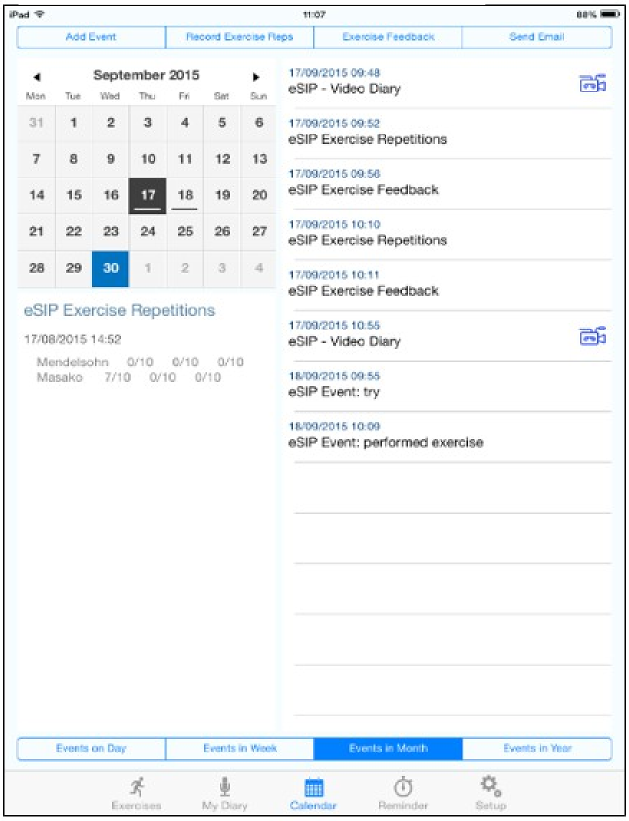

Figure A4. Exercise Repetitions

Figure A5. Exercise Feedback

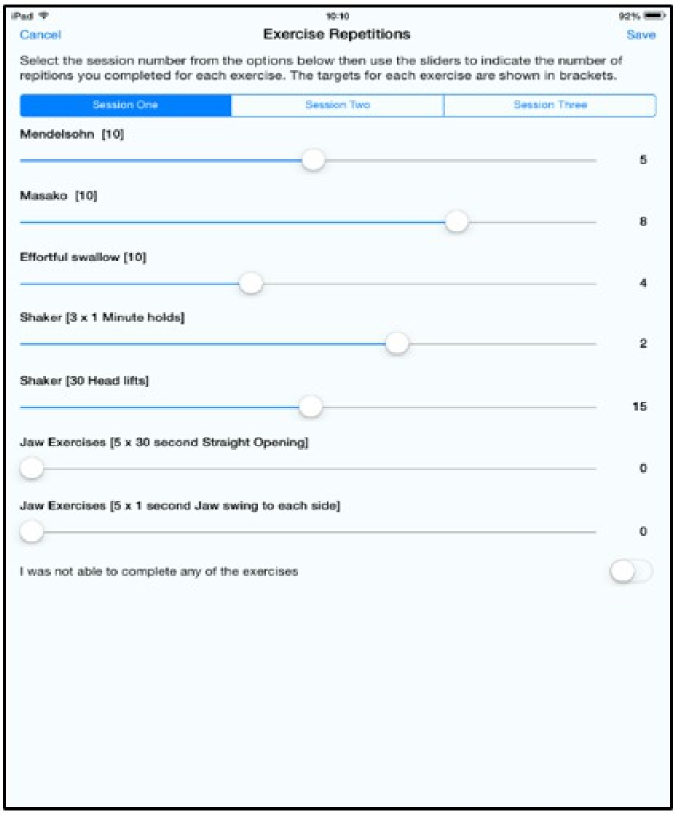
iPad
10:10
92%
Cancel
Exercise Repetitions
Save
Select the session number from the options below then use the sliders to indicate the number of repitions you completed for each exercise. The targets for each exercise are shown in brackets.
Session One
Session Two
Session Three
Mendelsohn [10]
5
Masako [10]
8
Effortful swallow [10]
4
Shaker [3 x 1 Minute holds]
2
Shaker [30 Head lifts]
15
Jaw Exercises [5 x 30 second Straight Opening]
0
Jaw Exercises [5 x 1 second Jaw swing to each side]
0
I was not able to complete any of the exercises

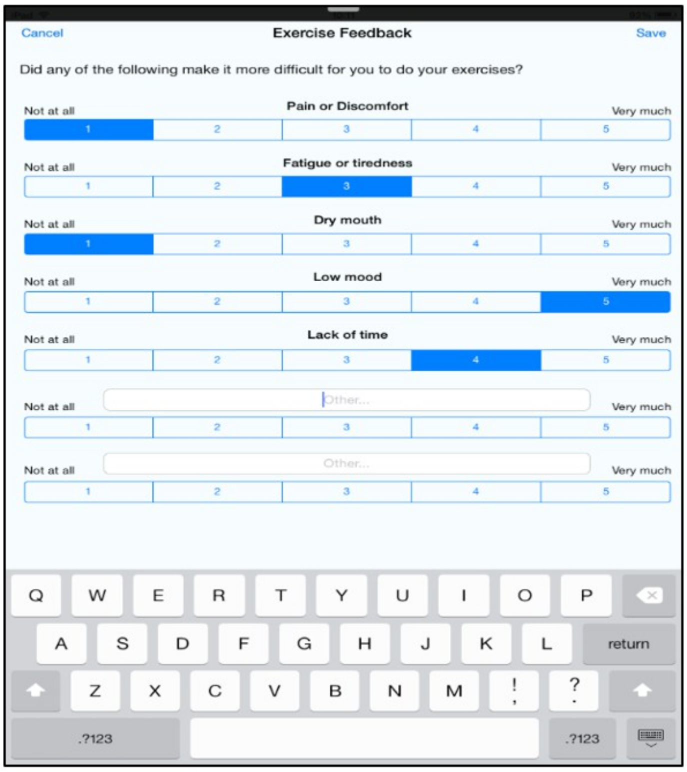
Cancel
Exercise Feedback
Save
Did any of the following make it more difficult for you to do your exercises?
Not at all
Pain or Discomfort
Very much
1 2 3 4 5
Not at all
Fatigue or tiredness
Very much
1 2 3 4 5
Not at all
Dry mouth
Very much
1 2 3 4 5
Not at all
Low mood
Very much
1 2 3 4 5
Not at all
Lack of time
Very much
1 2 3 4 5
Not at all
Other...
Very much
1 2 3 4 5
Not at all
Other...
Very much
1 2 3 4 5
Q W E R T Y U I O P
A S D F G H J K L return
Z X C V B N M ! ?
.?123
.?123
